# A systematic review of PET and PET/CT in oncology: A way to personalize cancer treatment in a cost-effective manner?

**DOI:** 10.1186/1472-6963-10-283

**Published:** 2010-10-08

**Authors:** Astrid Langer

**Affiliations:** 1 Institute of Health Economics and Health Care Management, Munich School of Management, Ludwig-Maximilians-Universität München, Munich, Germany; 2 Institute of Health Economics and Health Care Management, Helmholtz Zentrum München, Neuherberg, Germany

## Abstract

**Background:**

A number of diagnostic tests are required for the detection and management of cancer. Most imaging modalities such as computerized tomography (CT) are anatomical. However, positron emission tomography (PET) is a functional diagnostic imaging technique using compounds labelled with positron-emitting radioisotopes to measure cell metabolism. It has been a useful tool in studying soft tissues such as the brain, cardiovascular system, and cancer. The aim of this systematic review is to critically summarize the health economic evidence of oncologic PET in the literature.

**Methods:**

Eight electronic databases were searched from 2005 until February 2010 to identify economic evaluation studies not included in previous Health Technology Assessment (HTA) reports. Only full health economic evaluations in English, French, or German were considered for inclusion. Economic evaluations were appraised using published quality criteria for assessing the quality of decision-analytic models. Given the variety of methods used in the health economic evaluations, the economic evidence has been summarized in qualitative form.

**Results:**

From this new search, 14 publications were identified that met the inclusion criteria. All publications were decision-analytic models and evaluated PET using Fluorodeoxyglucose F18 (FDG-PET). Eight publications were cost-effectiveness analyses; six were cost-utility analyses. The studies were from Australia, Belgium, Canada, France, Italy, Taiwan, Japan, the Netherlands, the United Kingdom, and the United States. In the base case analyses of these studies, cost-effectiveness results ranged from dominated to dominant. The methodology of the economic evaluations was of varying quality. Cost-effectiveness was primarily influenced by the cost of PET, the specificity of PET, and the risk of malignancy.

**Conclusions:**

Owing to improved care and less exposure to ineffective treatments, personalized medicine using PET may be cost-effective. However, the strongest evidence for the cost-effectiveness of PET is still in the staging of non-small cell lung cancer. Management decisions relating to the assessment of treatment response or radiotherapy treatment planning require further research to show the impact of PET on patient management and its cost-effectiveness. Because of the potential for increased patient throughput and the possible greater accuracy, the cost-effectiveness of PET/CT may be superior to that of PET. Only four studies of the cost-effectiveness of PET/CT were found in this review, and this is clearly an area for future research.

## Background

Positron emission tomography (PET) is a three-dimensional diagnostic imaging technology in nuclear medicine measuring physiological function by looking at various functions of the body. It is a non-invasive diagnostic imaging tool using chemical neurotransmitters such as dopamine in Parkinson's disease or radiopharmaceuticals such as the radiolabelled glucose analogue Fluorodeoxyglucose F18 (FDG) in oncology.

PET was introduced in the 1970 s [1]. Intensive research activities in various PET applications gradually evolved to its clinical use first in neuropsychiatric disorders and cardiology, then in oncology. Oncology is now the most important application of PET. In oncology, PET can be used for [2]:

• Tumour detection and differential diagnosis of benign and malignant tumours

• Tumour staging and prognostic stratification

• Evaluation of treatment response

• Restaging and detection of recurrent cancer

• Radiation treatment planning

• Development of new anticancer drugs

Furthermore, PET can have different places in the diagnostic pathway [3]: at the beginning of the pathway as a triage, at the end of the pathway as an add-on, or as a replacement for an existing diagnostic procedure in the pathway.

The most recent innovation in PET scanners is the dual-modality PET/CT. By combining radiological (CT) and nuclear medicine (PET) imaging modalities, it is possible to add anatomical to functional information.

This study presents a systematic review of the cost-effectiveness of PET for the diagnosis and management of cancer. The aim of this systematic review is not to assess the clinical effectiveness of PET in oncology, but to appraise the cost-effectiveness of PET imaging in cancer management compared with non-invasive and invasive diagnostic modalities. After reading this systematic review, the reader should be able to: (1) describe oncologic PET and PET/CT indications for which economic evidence has already been shown; (2) illustrate the difficulties of evaluating the cost-effectiveness of innovative health technologies such as PET and PET/CT within the scope of the full diagnostic and treatment pathway; and (3) recognize the necessity for future prospective trial-based economic evaluation studies of oncologic PET and PET/CT. For more information on the clinical effectiveness of PET, the interested reader is referred to the Health Technology Assessment (HTA) report conducted by Facey et al. [4], which gives a detailed overview of the clinical effectiveness of FDG-PET in various cancers. Recommendations for the use of FDG-PET in oncology have also been published by Fletcher et al. [5]. Recently, an article regarding evidence (diagnostic effectiveness and cost-effectiveness) and methodological approaches for conducting economic evaluations of oncologic PET and PET/CT has been published by Buck et al. [2]. However, this article is not a systematic review and does not report on the specific characteristics of decision-analytic models assessing the cost-effectiveness of PET and PET/CT in oncology.

## Methods

Only full health economic evaluations (cost-effectiveness, cost-utility, cost-benefit analyses) comparing a PET-based strategy with different invasive and non-invasive diagnostic strategies in the clinical work-up of cancer patients were considered for inclusion. Furthermore, only fully published economic evaluations in English, French, or German were included. Economic evaluations included in two previous HTA reports of PET [6,7] were excluded to avoid duplication of efforts in reviewing and synthesizing evidence. A list of excluded studies on economic evaluation assessment with reasons for exclusion is provided in Table 1.

**Table 1:** List of excluded studies on economic evaluation assessment with reason for exclusion

Reference	Reason for exclusion
1. Barnett et al. 2010 [44]	Cost study

2. Basu, Rubello 2008 [45]	Review

3. Biersack 2009 [46]	Review

4. Bunyaviroch, Coleman 2006 [47]	Review

5. Buscombe, O'Rourke 2007 [48]	Review

6. Chua et al. 2008 [49]	Review

7. Chuck et al. 2005 [50]	Cost study

8. Cleemput et al. 2005 [6]	Health technology assessment report

9. Cleemput et al. 2008 [51]	Methodology paper

10. De Geus-Oei et al. 2006 [52]	Review

11. Devaraj et al. 2007 [53]	Review

12. Eloubeidi et al. 2005 [54]	Cost study

13. Facey et al. 2007 [4]	Health technology assessment report

14. Goh 2006 [55]	Comment

15. Gould 2006 [41]	Review

16. Graham 2009 [56]	Comment

17. Hayashi et al. 2005 [28]	Research question

18. Herbertson et al. 2009 [57]	Review

19. Heinrich et al. 2005 [58]	Cost study

20. Herder et al. 2006 [59]	Cost study

21. Hoekstra et al. 2006 [60]	Methodology paper

22. Juweid, Cheson 2006 [61]	Review

23. Krug et al. 2009 [62]	Cost study

24. Krug et al. 2008 [63]	Cost study

25. Krug et al. 2007 [64]	Methodology paper

26. Kwee et al. 2008 [65]	Review

27. Mattar 2007 [66]	Review

28. Meyers et al. 2006 [67]	Research question

29. Moulin-Romsee et al. 2008 [68]	Cost study

30. Nosotti et al. 2008 [69]	Cost study

31. Pertile 2009 [70]	Methodology paper

32. Pertile et al. 2009 [71]	Cost study

33. Plathow et al. 2008 [72]	Cost study

34. Poulou et al. 2009 [73]	Cost study

35. Remonnay et al. 2009 [74]	Cost study

36. Remonnay et al. 2008 [75]	Cost study

37. Rowan 2008 [76]	News

38. Sloka, Hollett 2005 [77]	Review

39. Spiro et al. 2008 [78]	Review

40. Strobel et al. 2007 [79]	Cost study

41. Sun et al. 2008 [80]	Review

42. Takahashi et al. 2007 [81]	Review

43. Uyl-de Groot et al. 2010 [82]	Cost study

44. Van den Bruel et al. 2007 [83]	Methodology paper

45. Van Tinteren et al. 2006 [84]	Comment

46. Van Hooren et al. 2009 [85]	Cost study

47. Von Schulthess et al. 2006 [27]	Review

48. Watson et al. 2006 [86]	Review

49. Yap et al. 2005 [87]	Cost study

50. Yasunaga 2009 [88]	Outcome evaluation study

51. Yasunaga et al. 2006 [89]	Outcome evaluation study

52. Zubeldia et al. 2005 [90]	Cost study

The following databases were searched from January 2005 to February 2010 for relevant economic evaluations concerning the use of PET imaging in oncology: Cochrane Library, DARE, EMBASE, HTA Database, NHS EED, PubMed, RePEc, and Web of Science. The search strategies used text words and corresponding indexing terms to capture all relevant studies. In Additional file 1, the full search strategies are provided.

As this literature review of PET was not restricted to FDG-PET, the search strategy developed by Mijnhout et al. [8] for a comprehensive search of the FDG-PET literature was not used. In addition to the electronic database searches, the internet was searched by Google and Google Scholar. Furthermore, citation tracking was performed using Google Scholar, and a manual search of the reference lists of recent reviews and included publications was undertaken.

Several reliable, comprehensive, and user-friendly checklists are available to assess the quality of health economic evaluations. The most widely used is the checklist of Drummond and Jefferson developed by the BMJ Economic Evaluation Working Party for the British Medical Journal [9]. However, the BMJ checklist does not provide detailed coverage of several key issues relevant to decision-analytic models such as model type, structural assumptions, cycle length, and health states [10]. Thus, to guide the quality assessment of the models used in the economic evaluations of PET, the quality assessment tool for decision-analytic models established by Philips et al. [11] was applied by two independent reviewers. This checklist covers three key dimensions of study quality: structure, data, and consistency. In this review, each item in the checklist had four possible responses: 1 for 'yes', 0 for 'no/not reported', ? for 'unclear', and NA for 'not applicable'. A summary score was not applied because a quality scoring system was not considered to be sufficiently reliable and valid as a means of quality assessment [12]. The appraisal of economic evidence favouring or opposing the use of PET for patients with cancer was based on the three key elements of the checklist established by Philips et al. [11]: structure, data, and consistency. The economic evidence was appraised as limited if one or more of these key elements were not fulfilled. By using this checklist, the validity of included studies, i.e. the risk of bias in individual studies, could also be assessed.

For the data collection process, a data extraction sheet was developed (based on the Centre for Reviews and Dissemination's data extraction template). The data extraction sheet for each study is available from the author on request. In Additional files 2 and 3, a summary of the considered data items can be found. Information was extracted from each included study on: (1) cancer/management decision; (2) author, year, country; (3) comparison; (4) patient group (base case); (5) measure of effectiveness; (6) incremental analysis; (7) method, perspective; (8) effectiveness (per patient); (9) cost (per patient); (10) incremental cost-effectiveness; and (11) sensitivity analysis.

## Results

### Overview of economic evaluations of oncologic PET

**Figure 1:**
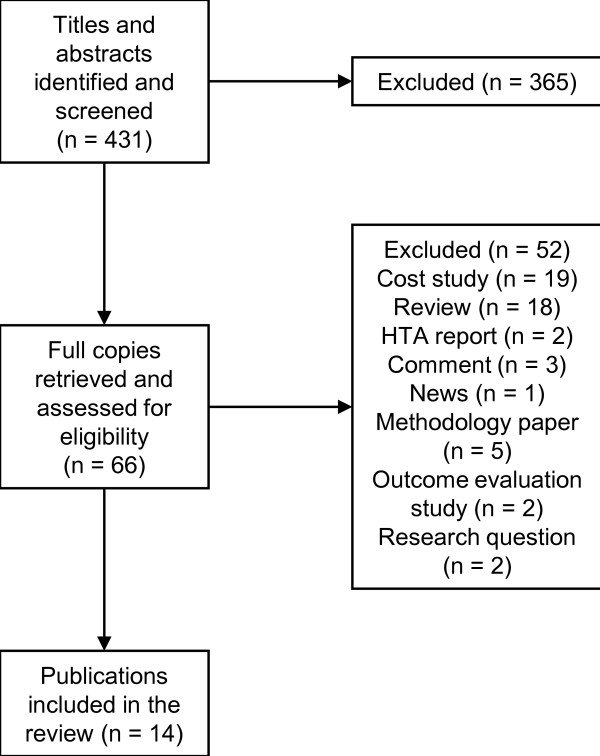
**Flowchart for selection of economic evaluation studies**.

From the systematic database search, 431 publications were identified. The full text of articles was investigated if the health technology appeared to be PET from the title and abstract and if quantitative economic results were reported. In all, 66 full copies were retrieved and assessed for eligibility. Finally, 14 publications met the inclusion criteria for this review, all of which were model-based economic evaluations and published in the English language: diagnosis of a solitary pulmonary nodule [13], staging of recurrent ovarian cancer [14], staging of liver metastases from colorectal cancer [15], staging of pulmonary metastases from malignant melanoma [16], staging of recurrent nasopharyngeal carcinoma [17], staging of head and neck cancer [18], staging of breast cancer [19], follow-up of non-small cell lung cancer (NSCLC) [20], and staging of NSCLC [21-26]. No further study was retrieved by citation tracking. A flowchart for the selection of economic evaluation studies can be found in Figure 1.

Eight publications were cost-effectiveness analyses; six were cost-utility analyses. The studies were from Australia, Belgium, Canada, France, Italy, Taiwan, Japan, the Netherlands, the United Kingdom, and the United States. In the base case analyses of these studies, cost-effectiveness results ranged from dominated to dominant. In Additional files 2 and 3, the characteristics of the economic evaluation studies and their main results are presented.

All publications provide an economic evaluation on a model basis. All modelling studies evaluated FDG-PET, i.e. PET using FDG as the radiotracer. Several publications built upon, revised, or extended previous decision models. Some articles provided new decision models. One study converted an existing decision tree to a Markov model [25]. Von Schulthess et al. [27] suggest that the cost-effectiveness of integrated PET/CT is superior to that of PET alone in some indications, because of both the higher diagnostic accuracy of integrated PET/CT and the potential for higher patient throughput. Only four studies [14,16,18,20] evaluated the cost-effectiveness of this new diagnostic imaging modality.

### Staging of breast cancer

Only one study could be identified that assessed the cost-effectiveness of PET for the preoperative axillary staging of breast cancer. In their model-based economic evaluation, Sloka et al. [19] compared PET and axillary lymph node dissection (ALND) in selected patients with ALND in all patients. A cost-effectiveness analysis of these two diagnostic strategies was undertaken using decision tree modelling to estimate the costs and effects for each strategy. The time horizon of the study was from the initial diagnostic studies to the final treatment of all treatment modalities (e.g. radiation therapy or modified radical mastectomy). The perspective adopted in the economic analysis was that of the hospital. The base case was defined as a 55-year-old woman with stage I or II breast cancer. Included in this cost-effectiveness analysis were the costs of diagnostics (PET, ALND) and the costs of treatment (chemotherapy, radiotherapy, breast-conserving surgery, and modified radical mastectomy). The PET strategy was strictly dominating, as it incurred lower costs and resulted in an increase in life expectancy. This result was relatively robust to sensitivity analysis. The authors found that PET for staging breast cancer may benefit patients in terms of an increase in life expectancy, and the hospital in terms of reduced costs. Owing to methodological deficiencies, the study quality is considered to be limited. In particular, the meta-analysis performed by the authors was not of high methodological quality. Furthermore, overhead costs, palliative care costs, and costs associated with quality of life were not considered. In addition, the authors did not compare their findings with those from other studies, and the use of probabilistic sensitivity analysis would have captured the issue of uncertainty in the model parameters better.

### Staging of liver metastases from colorectal cancer

Only one study was found that evaluated the cost-effectiveness of PET in the management of patients with metachronous liver metastases after curative resection of colorectal cancer. Lejeune et al. [15] used a decision tree model to compare two diagnostic strategies: CT versus CT followed by PET. The base case was defined as a 68-year-old patient with suspected metachronous liver metastases detected by ultrasonography during follow-up. The economic analysis was carried out from the perspective of the health care system and the time horizon of the model was the patient's lifetime. The costs of diagnostic tests (CT, magnetic resonance imaging (MRI), PET, and liver biopsy) and treatment procedures (exploratory surgery, surgery, and palliative treatment) were included. As CT followed by PET was both more effective and less costly than CT alone, it was found to be the dominant strategy. Lejeune et al. concluded that CT+PET was as effective as CT alone in terms of life expectancy, but less expensive mainly because of cost savings resulting from a decrease in inappropriate surgeries. The sensitivity analysis showed the robustness of the model. Overall, this study was found to be of good methodological quality.

### Staging of pulmonary metastases from malignant melanoma

Only one study could be retrieved that assessed the cost-effectiveness of PET/CT in staging pulmonary metastases from malignant melanoma. Krug et al. [16] used a Markov model over a 10-year period to compare two different surveillance programmes in patients with suspected pulmonary metastases from malignant melanoma: PET/CT or whole-body CT. The study was carried out from the perspective of the health care system. The following costs were included in the analysis: screening (visit, blood sampling and chest X-ray), surgery and complications, chemotherapy and complications, palliative treatment, PET/CT, and CT. The PET/CT strategy was strictly dominating, as it incurred lower costs and resulted in an increase in life-months gained. The authors concluded that integrating PET/CT in the management of patients with high-risk malignant melanoma appeared to be less costly and more accurate by avoiding 20% of futile surgeries as well as by providing a small survival benefit at 10 years. In this study, the issue of data identification was not appropriately addressed, and the rationale for the model structure was unclear. However, the issue of uncertainty was satisfactorily addressed using a probabilistic sensitivity analysis, the findings of which were extensively illustrated and described.

### Staging of recurrent nasopharyngeal carcinoma

Since 2005, the cost-effectiveness of PET in the staging of recurrent nasopharyngeal carcinoma has only been assessed by Yen et al. [17]. They used a decision tree model to evaluate the cost-utility of PET in detecting local recurrences of nasopharyngeal cancer after treatment. The base case consisted of a 46-year-old male with suspected recurrent nasopharyngeal cancer at follow-up. In their study, three different diagnostic strategies were compared: MRI only, PET only, and PET after an uncertain result from MRI (MRI-PET strategy). The economic analysis only included the costs of diagnostic tests (MRI and PET). The incremental cost per quality-adjusted life-year (QALY) gained over MRI was US$1,389 for PET and US$462 for MRI-PET. The results were sensitive to the probability of uncertain MRI and the cost ratio of PET to MRI. Yen et al. concluded that MRI followed by PET was the most cost-effective strategy, but PET alone could become the preferred strategy on account of the cost of PET decreasing at a faster rate than the cost of MRI. However, the authors' conclusions should be considered with caution, because there were several limitations to their economic analysis. First, the two imaging techniques were not described well, and it is not clear why the MRI alone strategy was considered for inclusion, because it was found to be ineffective in detecting recurrent nasopharyngeal carcinoma. The perspective and time horizon of the model were not reported. Only the costs of MRI and PET were included in the economic analysis, so other direct health resources, which were not included, might have influenced the overall study findings. The price year was not reported, and the discounting of costs and health benefits would have been appropriate as the time horizon seemed to be the patient's lifetime. Furthermore, the utility values were based on the Visual Analogue Scale being commonly considered to be inferior to the standard gamble or time trade-off methods. Finally, the issue of uncertainty was not appropriately addressed as only univariate and multivariate sensitivity analyses were carried out on a few parameters, and the validity of the data sources was not reported.

### Staging of head and neck cancer

Only one study reported the cost-effectiveness of PET/CT for staging head and neck cancer. Sher et al. [18] assessed the cost-effectiveness of PET/CT as a predictor of the need for adjuvant neck dissection (ND) compared with ND for all patients. A Markov model was developed to describe health states in the 5 years after chemoradiotherapy in a 50-year-old man with node-positive stage IVA (i.e. T1-3 N2 M0) squamous cell carcinoma of the oropharynx. The following three strategies were compared: dissect all patients, dissect patients with residual disease (RD) on CT, and dissect patients with RD on PET/CT. The costs of diagnostics (CT and PET/CT) and treatment (immediate ND, salvage ND, salvage surgery for local recurrence, chemotherapy for metastasis, hospice care) were included. ND for patients with RD on PET/CT was found to be the dominant strategy. The model has some limitations, and thus should be considered with caution. The perspective of the model was not stated, and the primary decision-maker was not specified. Furthermore, the data sources used to develop the structure of the model were not specified. Concerning the key theme of data, the issue of data identification and the assessment of uncertainty were not appropriately addressed, because data identification methods were not reported in detail, and methodological and structural uncertainty and systematic differences between patient subgroups were not considered.

### Staging of non-small cell lung cancer

Several studies evaluated the cost-effectiveness of PET for staging NSCLC. To determine the cost-effectiveness of PET for mediastinal staging of potentially operable NSCLC from the health care system perspective, Alzahouri et al. [21] developed a decision tree model. Four diagnostic strategies were compared: CT only, PET for negative CT, PET for all patients with anatomical CT, i.e. PET for the staging decision and CT for anatomic location of mediastinal nodes whereas size was ignored, and CT plus PET for all patients. The base case was defined as a 65-year-old patient, in whom NSCLC had been histologically established and assessed as operable. The time horizon was not explicitly stated, but seemed to be the patient's lifetime. The following costs were included: CT, PET, cervical mediastinoscopy, surgery, and chemoradiation. In comparison with CT only, which was used as the baseline strategy, PET for all with anatomical CT was found to be dominant, whereas the CT and PET for all strategy was found to be dominated by the baseline strategy. The incremental cost-effectiveness ratio (ICER) of PET for negative CT versus CT alone was €33,165 per life-year gained. Sensitivity analyses showed the robustness of the results. Alzahouri et al. concluded that PET for all with anatomical CT was the most cost-effective strategy. In this study, the primary decision-maker was not specified, and the rationale for the model structure was less clear. Further, the time horizon of the model was not explicitly stated, and the issue of data identification was not appropriately addressed. However, Alzahouri et al. compared their results extensively with those from other studies.

Bird et al. [24] used a decision model to evaluate the cost-effectiveness of PET in the management of potentially operable patients with NSCLC. Two diagnostic strategies were compared: conventional work-up (CWU), consisting of an X-ray, a chest CT scan, and bronchoscopy, followed by whole-body PET versus CWU alone. These two diagnostic strategies were applied to two subgroups of NSCLC patients: patients with a negative CT scan (CT-negative patients) and patients with a positive CT scan (CT-positive patients). The cost of diagnostic tests (PET, mediastinoscopy) and the cost of treatment (surgery, radiotherapy, and chemotherapy) were included. The incremental cost per QALY gained for the CT-negative strategy was A$14,581 and A$52,039 for the CT-positive strategy. Bird et al. concluded that there was much uncertainty surrounding the base case analysis, particularly in CT-positive patients. In this study, the issue of data identification was appropriately addressed. Bird et al. provided a detailed summary table of the studies found in their literature review. There were three cost-utility analyses [26,28,29], three cost-effectiveness analyses [22,30,31], and two comparisons of costs and effects [32,33], in addition to two randomized controlled trials [34,35]. Most studies suggested that PET is likely to be cost-effective, particularly in CT-negative patients. Further, in this study, the issue of uncertainty was extensively illustrated and described. However, a graphical representation of the model structure was not provided. Finally, the authors stated that the perspective was societal but, given the perspective assumed, not all relevant costs were included.

Kee et al. [25] converted an existing decision tree model of the impact of PET on preoperative staging of NSCLC to a Markov model to include patient-elicited utilities. Based on the Markov model, the expected value of perfect information (EVPI) associated with three sources of uncertainty (the accuracy of PET, the accuracy of CT, and the patient-related utility of a futile thoracotomy) was estimated. The following costs were included: PET, mediastinoscopy, surgery, radical radiotherapy, chemotherapy, and best supportive care. The ICER of the strategy with PET versus the strategy without PET was £6,704 for a 50-year-old, £8,385 for a 60-year-old, £10,636 for a 70-year-old, and £13,785 for an 80-year-old. The model confirmed the cost-effectiveness of PET and showed that the EVPI associated with the utility of futile thoracotomy exceeded that associated with measures of diagnostic accuracy. This study has several limitations. The perspective of the analysis was not reported. The rationale for the model structure, the statement of scope/perspective, and the structural assumptions were not clear. Another limitation concerns the issue of data identification. Further, the four principal types of uncertainty were not all addressed.

To evaluate the cost-effectiveness of the introduction of PET in the clinical management of lung cancer patients, Mansueto et al. [23] compared three different diagnostic strategies: CT alone, PET for indefinite CT, and PET for all. For each of these strategies, expected costs and benefits, as measured by life-years gained, were estimated using decision tree models. The perspective of the health care system was adopted in the study. The cost of diagnostic tests and surgical procedures were included. When compared with CT alone, the additional costs per life-year gained were €2,508 when using PET for indefinite CT and €415 when using PET for all. In the sensitivity analyses, PET for all remained the most cost-effective strategy. The authors stated that their results generally agreed with those from other economic evaluations [22,30,31]. However, neither the rationale for the model structure nor the structural assumptions were sufficiently clear. The time horizon of the model was not reported. Further, the issue of data identification and the issue of uncertainty were not appropriately addressed.

The modelling study commissioned by NICE [26] was built upon the Health Technology Board for Scotland (HTBS) and Dietlein models [29,31]. The authors considered two groups of patients who were expected to benefit most from PET. The first group were patients with normal-sized lymph nodes on CT (i.e. negative CT results) being considered for PET (surgery model). The second group consisted mainly of those patients with enlarged nodes on CT (i.e. positive CT results) who were being considered for radical radiotherapy (radiotherapy model). In the surgery model, three strategies were considered. In the first strategy, patients went straight to thoracotomy. In the second strategy, the patients had a mediastinoscopy and then received either radical radiotherapy (N2/3) or thoracotomy (N0/1). In the third strategy, patients had a PET scan and then received either active supportive care (M1), thoracotomy (N0/1, M0), or mediastinoscopy (N2/3, M0). The authors found that the mediastinoscopy strategy was dominated by the PET strategy. Compared with the thoracotomy strategy, the PET strategy resulted in 22% fewer futile thoracotomies, 1% fewer surgical deaths, and a better selection of patients for radical radiotherapy. This resulted in an increase of 0.04 QALYs per patient. The ICER of the PET strategy compared with the thoracotomy strategy was £7,200 per QALY gained. Sensitivity analyses showed that the base case result was not sensitive to any single parameter other than unit costs. In the radiotherapy model, only two strategies were evaluated. The first strategy was that all patients go straight to radiotherapy, and the second strategy was that all patients have a PET scan and then receive active supportive care (M1), thoracotomy (N0/1), or radical radiotherapy (N2/3). In comparison with the radiotherapy strategy, the PET strategy resulted in less futile radiotherapy and some patients benefiting from curative surgery. However, some patients had unnecessary surgery, and some missed radical radiotherapies. The estimated additional cost was £9,489 per QALY gained. As in the surgery model, the decision to opt for PET was not sensitive to any single parameter other than unit costs. The authors compared their results extensively with those from the HTBS model [29] and the PLUS study [34], and explained any differences between them. Although the publication was commissioned by NICE, the primary decision-maker was not specified. Further, the selection of the comparators was not explicitly justified, and the issue of uncertainty was not appropriately addressed.

In the cost-effectiveness analysis by Nguyen et al. [22], the objective was to evaluate the cost-effectiveness of PET in the management of potentially operable NSCLC in Quebec, Canada. A decision tree model was developed to predict the costs and effects of using PET scanning as a means of detecting mediastinal and distant metastases. Two diagnostic strategies were compared: CT alone and initial CT followed by whole-body PET. The viewpoint adopted in the economic analysis was that of the health care system. Only the direct costs of the health care system were included in the economic analysis. The ICER was C$4,689 per life-year gained. In the univariate sensitivity analysis, the ICER of CT plus PET ranged from C$3,000 to C$5,000 per life-year gained. If the threshold ICER was set at C$50,000 per life-year gained, the authors found that 95% of the Monte Carlo simulations would be below this threshold. The authors reported that their findings were similar to those from other studies. Nguyen et al. concluded that PET is an intervention that requires an acceptable investment for each life-year gained. Overall, the study was of good methodological quality.

### Follow-up of non-small cell lung cancer

Only one study reported on the cost-effectiveness of PET/CT in the follow-up of NSCLC. The cost-utility analysis by van Loon et al. [20] was based on a Markov model, with a hypothetical cohort of NSCLC patients treated with curative radiotherapy with or without chemotherapy, and a 5-year time horizon. The study objective was to assess the long-term cost-effectiveness of three follow-up strategies with different imaging modalities 3 months after therapy: PET/CT-based follow-up, chest CT-based follow-up, and conventional follow-up with a chest X-ray. Additionally, a strategy of performing a PET/CT scan only in asymptomatic patients was considered. Asymptomatic patients were defined as patients with no symptoms 3 months after treatment. The perspective of the health care system was adopted in the study. The costs of diagnostic tests (initial procedure and subsequent follow-up), radical treatment (adrenal resection and radical radiotherapy), palliative radiotherapy, palliative chemotherapy, and death (cancer-related terminal care or death from other causes) were included in the economic analysis. The incremental cost per QALY gained compared with conventional follow-up was €264,033 for CT and €69,086 for PET/CT. For PET/CT in the subgroup of asymptomatic patients, the ICER was €42,265 per QALY gained. In the whole study group, the probabilistic sensitivity analysis showed that there was considerable uncertainty regarding the optimal follow-up strategy. At a ceiling ratio of €80,000 per QALY, the PET/CT-based follow-up in asymptomatic patients only had the highest probability of being cost-effective (73%). The authors found that the PET/CT-based follow-up was potentially cost-effective and was economically more attractive than the CT-based follow-up, especially in the subgroup of asymptomatic patients. Overall, the study used robust methodology. However, the reporting of some data sources was limited, and the internal and external validity of the study was not explicitly investigated.

### Staging of recurrent ovarian cancer

Only one study could be retrieved that assessed the cost-effectiveness of PET/CT in staging recurrent ovarian cancer. Mansueto et al. [14] evaluated the economic impact of the introduction of PET/CT in the early detection of suspected ovarian cancer recurrence based on a decision tree model. Clinical data used in the economic evaluation were taken from Mangili et al. [36]. The measure of benefits used in the economic analysis was the number of surgical procedures avoided. The study population consisted of 32 patients. Three different diagnostic strategies were compared: CT only, PET/CT for negative CT, and PET/CT for all patients. In comparison with CT alone, the PET/CT for all strategy resulted in an ICER of €227 per surgery avoided. PET/CT for negative CT was found to be dominated by CT only, because the first was more expensive, but less effective. Mansueto et al. concluded that the introduction of PET/CT was cost-effective and changed the clinical management of patients with suspected ovarian cancer recurrence towards more appropriate treatment. Especially because of the small sample size, the study results should be considered with caution. Further, the rationale of the model structure was not established well, and the issues of data identification and assessment of uncertainty were not appropriately addressed.

### Diagnosis of a solitary pulmonary nodule

Since 2005, the cost-effectiveness of PET in diagnosing solitary pulmonary nodules has only been evaluated by Lejeune et al. [13]. In their cost-effectiveness analysis, a decision tree model was used to compare the cost-effectiveness of PET with that of standard imaging modalities in managing solitary pulmonary nodules. The diagnostic strategies evaluated were: wait and watch (WW), PET and anatomical CT (PET strategy), i.e. CT only to define the location, and CT followed by PET (CT+PET). The base case was defined as a 65-year-old man currently smoking 1.5 packs per day, with a 2-cm solitary pulmonary nodule (SPN) without calcification, specula and enlargements of mediastinal lymph nodes, and a malignancy risk of 43%. The costs of diagnostic tests (radiography, CT, PET, and transthoracic needle biopsy) and the costs of surgical procedures (video-assisted thoracic surgery, wedge resection, and lobectomy) were included. From the French health care system perspective, the incremental cost per life-year gained over WW was €4,790 when using PET and €3,022 when using CT+PET. In comparison to the PET strategy, CT+PET was found to be the dominant strategy. The results of the sensitivity analysis showed that the CT+PET strategy remained the most cost-effective strategy when the risk of malignancy was between 5.7% and 87%, whereas WW was more cost-effective between 0.3% and 5%. The authors concluded that CT+PET was cost-effective in diagnosing a solitary pulmonary nodule in patients with a malignancy risk of at least 5.7%. This study was found to be of good methodological quality.

## Discussion

### Summary of economic evidence for PET and PET/CT in oncology

The technology of PET can be applied to different indications in oncology. At present, limited evidence is available on the cost-effectiveness of PET in the staging of breast cancer, liver metastases from colorectal cancer, pulmonary metastases from malignant melanoma, recurrent nasopharyngeal carcinoma, head and neck cancer, or recurrent ovarian cancer, and in the follow-up of NSCLC. Hence, no sound conclusions can be drawn on the cost-effectiveness of PET and PET/CT in the diagnostics of these indications. Since 2005, the most extensive evidence has been provided by six studies on the cost-effectiveness of PET in staging NSCLC.

### Methodology of the economic evaluations

As shown in Additional files 4 and 5, the evaluations were of varying methodological quality, with some deviating from economic evaluation standards. For instance, in several studies, the perspective of the model was not stated, and in none of the studies was the primary decision-maker clearly specified, although one publication [26] was commissioned by NICE.

In general, the quality of the results largely depends on the quality of the model and the quality of the underlying data. Issues covered under the key theme of data relate to data identification methods and the assessment of uncertainty. None of the studies addressed all four types of uncertainty. Probabilistic sensitivity analysis was only performed in six models. Most of the health economic evaluations relied on univariate sensitivity analysis. The sensitivity analyses revealed that cost-effectiveness was primarily influenced by the cost of PET, the specificity of PET, and the risk of malignancy. The issue of generalizability to other settings was partly addressed in the sensitivity analyses. As all studies identified were model-based economic evaluations, data identification is key for the quality of such economic evaluation studies. Table 2 provides an overview of the data sources used to populate the models.

**Table 2:** Overview of data sources used to populate the models

*Staging of breast cancer*		
Sloka et al. 2005 [19]	Data sources	**Accuracy data**: From literature (MEDLINE, Current Contents, EMBASE; completed in December 2003; details of literature search given); the authors conducted a meta-analysis using 12 published PET studies of staging axillary lymph node metastases; **Life expectancy**: From literature; life expectancies calculated by authors using the DEALE method; **Costs**: From literature; **Mortality**: Mortality estimated by authors; **Quality of life**: Not relevant.
	
	Dates to which data relate	The PET accuracy data were derived from studies published between 1989 and 2001. The price year was 2000.

*Staging of liver metastases from colorectal cancer*		

Lejeune et al. 2005 [15]	Data sources	**Accuracy data**: From literature, second liver biopsy sensitivity from expert opinion, PET accuracy data were taken from three published studies; **Life expectancy**: Burgundy Digestive Cancer Registry, life expectancies calculated by authors using the DEALE method; **Costs**: The costs of the diagnostic procedures were obtained from 'Nomenclature Générale des Actes Professionnels'. The cost of hospital stay required for liver biopsy and the cost of treatment procedures were based on the national reimbursement rate for the medical department of the Dijon University Hospital. The length of hospitalization for symptomatic treatment was estimated on the basis of the national hospital database on diagnosis-related groups. Other resource quantities were based on expert opinion; **Mortality**: From literature; **Quality of life**: Not relevant.
	
	Dates to which data relate	The PET accuracy data were derived from three studies published between 1997 and 2002. The price year was 2004.

*Staging of pulmonary metastases from malignant melanoma*		

Krug et al. 2010 [16]	Data sources	**Accuracy data**: From literature, PET accuracy data were taken from three published studies; **Life expectancy**: The life expectancies were calculated by running the model over different time periods and until the entire cohort died; **Costs**: The unit cost values of hospitalization, drugs, surgery, diagnostic procedures, and the direct health care-related out-of-pocket expenses of patients were obtained from the current public prices published by the RIZIV/INAMI (Health Insurance Institution in Belgium). A micro costing approach was used to calculate the true actual costs of performing a PET/CT study. Resource quantities were based on the resources used by a cohort of patients followed in standardized administrative databases of 19 hospitals between 2005 and 2006 identified by the Diagnosis Related Groups (DRG) codes; **Mortality**: From literature; **Quality of life**: Not relevant.
	
	Dates to which data relate	The PET accuracy data were taken from three studies published between 2006 and 2007. The price year was 2009.

*Staging of recurrent nasopharyngeal carcinoma (NPC)*		

Yen et al. 2009 [17]	Data sources	**Accuracy data**: From literature, probability of an uncertain MRI result estimated by authors, PET accuracy data were taken from four published studies; **Life expectancy**: From literature and from life tables from the Department of Health, Executive Yuan, Taiwan, 2002; Life expectancies calculated by authors using the DEALE method; **Costs**: Based on reimbursements from National Health Insurance, Taiwan; **Mortality**: From literature; **Quality of life**: Utilities elicited from 10 recurrent NPC patients and 10 oncologists involved in the management of these patients.
	
	Dates to which data relate	The PET accuracy data were taken from four studies published between 2002 and 2004. The price year was not reported.

*Staging of head and neck cancer*		

Sher et al. 2010 [18]	Data sources	**Accuracy data**: From literature, the PET accuracy data were taken from a meta-analysis of the role of PET in the follow-up of head and neck squamous cell carcinoma following radiotherapy or chemoradiotherapy; **Life expectancy**: The (quality-adjusted) life expectancy was calculated by running the model; **Costs**: Costs were taken from the published literature and publicly available Medicare payment schedules; **Mortality**: From literature; **Quality of life**: QALYs were calculated using utilities that were taken from literature or based on authors' assumptions.
	
	Dates to which data relate	The meta-analysis of PET was published in 2008. The price year was 2006.

*Staging of recurrent ovarian cancer*		

Mansueto et al. 2009 [14]	Data sources	**Accuracy data/Life expectancy/Mortality**: From Mangili et al. [36]; **Costs**: The costs were based on DRG tariffs; **Quality of life**: Not relevant.
	
	Dates to which data relate	The clinical study by Mangili et al. was published in 2007.The price year was 2006.

*Staging of non-small cell lung cancer (NSCLC)*		

Alzahouri et al. 2005 [21]	Data sources	**Accuracy data**: From literature, PET accuracy data were taken from the cost-effectiveness analysis of Dietlein et al. [31] and from a meta-analysis by Gould et al. [91]; **Life expectancy**: From the cost-effectiveness analysis by Dietlein et al.; **Costs**: PET and chemoradiation from literature, CT and cervical mediastinoscopy from 'Nomenclature Générale des Actes Professionnels', surgery from 'Echelle Nationale des Coûts'; **Mortality**: From literature; **Quality of life**: Not relevant.
	
	Dates to which data relate	The PET accuracy data were derived from two studies published between 2000 and 2003. The price year was not reported.

Bird et al. 2007 [24]	Data sources	**Accuracy data**: From literature, PET accuracy data were taken from the HTBS [29] and from the NCCAC model [26]; **Life expectancy**: Life expectancies were taken from the cost-effectiveness analysis by Dietlein et al. [31]; **Costs**: From literature and from the Australian National Hospital Cost Data Collection; the chemotherapy costs were estimated using the HTBS model, the Rosenthal study [92], and Australian data; **Mortality**: Mortality rates were taken from the cost-effectiveness analysis by Dietlein et al.; **Quality of life**: QALYs were based upon the values used in the HTBS model.
	
	Dates to which data relate	The PET accuracy data were derived from two HTA reports published between 2002 and 2005. The price year was 2006.

Kee et al. 2010 [25]	Data sources	**Accuracy data/Life expectancy/Costs/Mortality**: From the HTBS model [29]; **Quality of life**: Utilities elicited from 75 NSCLC patients.
	
	Dates to which data relate	The PET accuracy data were derived from a HTA report published in 2002. The price year was not reported.

Mansueto et al. 2007 [23]	Data sources	**Accuracy data**: From a meta-analysis by Gould et al. [91]; **Life expectancy**: From the cost-effectiveness analysis by Dietlein et al. [31]; **Costs**: From values of diagnosis-related groups and tariffs of the regional health care system; **Mortality**: From literature; **Quality of life**: Not relevant.
	
	Dates to which data relate	The accuracy data were taken from a study published in 2003. The price year was 2005/6.

National Collaborating Centre for Acute Care 2005 [26]	Data sources	**Accuracy data**: From the HTBS model [29]; **Life expectancy**: From the cost-effectiveness analysis by Dietlein et al. [31]; **Costs**: From the NHS reference costs 2002, from literature, or from individual NHS trusts; **Mortality**: From the cost-effectiveness analysis by Dietlein et al.; **Quality of life**: From the HTBS model.
	
	Dates to which data relate	The PET accuracy data were taken from the HTBS model published in 2002. The price year was 2002/3.

Nguyen et al. 2005 [22]	Data sources	**Accuracy data**: From Pieterman et al. [93]; **Life expectancy**: From literature; **Costs**: The costs of PET were derived from the costs of PET scanning at the Centre Hospitalier Universitaire de Sherbrooke, Quebec. The costs of hospital stay were determined by the Ministère de la Santé et des Services Sociaux's 1998-1999 diagnosis-related groups database. The figures for physicians' fees were taken from the Medical Specialists' Manual; **Mortality**: From literature; **Quality of life**: Not relevant.
	
	Dates to which data relate	The PET accuracy data were derived from a study published in 2000. The price year was 1998/99.

*Follow-up of non-small cell lung cancer (NSCLC)*		

Van Loon et al. 2010 [20]	Data sources	**Accuracy data**: Data on the ability of follow-up strategies to detect progression were taken from a previous prospective study [94]; **Life expectancy**: The (quality-adjusted) life expectancy was calculated by running the model; **Costs**: The costs of diagnostic procedures and treatment were taken from the Dutch Health Insurance Board. Other costs were based on the guideline costing statement provided by the UK National Institute for Health and Clinical Excellence (NICE) and a published randomized controlled trial; **Mortality**: From literature, supplemented by expert opinion; **Quality of life**: Utility values were taken from a published cross-sectional study, supplemented by expert opinion.
	
	Dates to which data relate	The prospective study was published in 2009. The price year was 2007.

*Diagnosis of a solitary pulmonary nodule (SPN)*		

Lejeune et al. 2005 [13]	Data sources	**Accuracy data**: From literature, the PET accuracy data were taken from five published studies; **Life expectancy**: From literature; **Costs**: The costs of radiography and CT were obtained from the 'Nomenclature Générale des Actes Professionnels'. The cost of PET was derived from two French studies. The costs of treatment procedures were derived from diagnosis-related groups for 2003 in the public health care sector. The cost of the patient's hospital stay was derived from the French national public cost scale, which was derived from a representative sample of French hospitals; **Mortality**: From literature; **Quality of life**: Not relevant.
	
	Dates to which data relate	The PET accuracy data were derived from different studies published between 1990 and 1998. The price year was not reported.

The level of reporting of the methods of deriving and identifying estimates of measures of effectiveness made it difficult to ascertain their validity, i.e. whether the best available evidence was used to populate the model. This selective reporting may result in a publication bias. A further problem was that the effectiveness data were often not derived from recently published studies, which will consequently not reflect current health technology use and practice.

The estimation of measures of health benefit was modelled using a decision tree or a Markov model, which was appropriate for the particular study question. All but one study [14] considered generic outcome measures that were directly related to the patients' health. In oncology, where survival is one of the primary objectives of interventions, life expectancy is a common outcome measure. However, the use of a cost-utility analysis using QALYs could capture the disutility of futile procedures and would permit comparisons with other health care interventions. Nguyen et al. [22] pointed out that, in the short term, it is implausible that PET would improve survival in cancer patients per se. Rather, its accuracy would reside in the ability to improve the patients' quality of life by avoiding unnecessary, debilitating interventions and providing the patients with quicker access to treatment. Six studies considered the impact of PET on quality of life [17,18,20,24-26].

Most studies used a different time horizon for costs and health benefits. Whereas health benefits could be incurred over the patient's lifetime, the costs were only incurred over a shorter time horizon. In none of the studies were the indirect costs included, although one economic analysis was carried out from a societal perspective [24]. Furthermore, details of discounting were often not provided, and costs and quantities were often not reported separately.

Finally, in several models, the ICER was not calculated correctly. The costs and effects of each strategy were compared with a baseline strategy as opposed to comparing each consecutively less effective strategy with the next most effective non-dominated strategy.

### Transferability of the results

Transferability relates to the extent to which the results obtained are relevant to different settings [10]. Methodological, healthcare system, and population characteristics may determine whether the results can be generalized [37]. Various groups of authors have suggested checklists for assessing the transferability of health economic evaluation results between countries, e.g. [37-40]. However, as these checklists almost exclusively relate to transferring the results of pharmacoeconomic evaluations, their use in assessing the transferability of economic evaluation studies in the field of medical technology can be questioned [39]. Furthermore, as diagnostic pathways can differ from country to country [6], the results of economic evaluations may hardly be transferable. Thus, the findings of economic evaluation studies are only relevant insofar as they represent current clinical practice in the specific decision context.

### Comparison with other reviews

This economic evaluation assessment augmented the systematic search undertaken in two previous HTA reports [6,7]. Thus, economic evaluations included in both these reports were excluded to avoid duplication of efforts in reviewing and synthesizing evidence. Furthermore, in this review, studies were limited to those assessing the cost-effectiveness of oncologic PET and PET/CT.

The HTA report conducted by Müller et al. [7] compared the cost-effectiveness of PET with common alternative diagnostic interventions to assess current economic evidence in the areas of oncology, cardiology, and neuropsychiatric disorders. Based on a broad systematic literature search, 14 oncological studies and 7 cardiological studies were considered for inclusion. No relevant publications were found in the area of neuropsychiatric disorders. The methodological quality of the oncological studies was judged to be good or very good. Based on the literature review, PET was considered to be cost-effective only in staging NSCLC. The authors pointed out the need for further economic evaluations based on qualitatively high-ranking clinical trials.

In their HTA report, Cleemput et al. [6] assessed the clinical effectiveness and cost-effectiveness of PET in oncology, cardiology, and neurology. Economic evaluations comparing PET with appropriate alternatives were searched for all these disease areas for which at least evidence of level 3 (diagnostic thinking efficacy) was available. Based on a systematic literature search, 22 health economic evaluations were identified and appraised using the BMJ checklist [9]. The methodology of the studies was found to be of varying quality. The authors pointed out that the economic evaluations were hardly comparable because of different diagnostic pathways, models, perspectives, and time horizons. They concluded that there was only evidence that the addition of PET to CT for staging NSCLC was cost-effective, although the incremental benefit in terms of life-years gained was small.

Table 3 provides a summary of the studies included in these two HTA reports, but only those studies are considered that meet the inclusion criteria of this review.

**Table 3:** Overview of economic studies included in two previous HTAs

Author, year, country	Study population*	Comparison alternatives	Perspective/type of study	Results and unit*	Economic evidence
*Recurrence of colorectal cancer*					

Park et al. 2001, USA [95]	Patients with an increase in carcinoembryonic antigen levels of > 5 ng/ml during follow-up testing after the resection of their primary CRC	CT+PET versus CT	Public payer (Medicare)/CEA using a model approach (decision tree)	ICER (US$/LYG): 16,437	Fair^b^

Sloka et al. 2004, Canada [96]	65-year-old patient presenting with suspected recurrent CRC	CT+PET versus CT	Hospital/CEA using a model approach (decision tree)	Cost savings: C$1,758^a^	Fair^b^

*Staging of head and neck cancer*					

Hollenbeak et al. 2001, USA [97]	HNSCC patients with no evidence of lymph node involvement	CT+PET versus CT	Hospital/CEA/CUA using a model approach (decision tree)	ICER (US$/LYG (US$/QALY)): 8,718 (2,505)	Fair^b^

*Restaging of malignant lymphoma*					

Bradbury et al. 2002, UK [29]	HD patients who have achieved a partial or complete response to induction therapy	(1) All for surveillance; (2) All for consolidation; (3) CT; (4) PET after positive CT; (5) (CT)+PET	Health care system/CUA using a model approach (decision model with two components: decision tree and Markov model)	Strategies 4 and 5 were found to be cost-effective, provided WTP exceeds £1000/LYG, and for almost all input values considered, provided WTP exceeds £5000/LYG	Good^b^

*Diagnosis of solitary pulmonary nodules*					

Dietlein et al. 2000, Germany [98]	62-year-old man with a SPN of up to 3 cm without calcification, specula and enlargement of mediastinal lymph nodes	(1) WW; (2) TNB; (3) Exploratory surgery; (4) PET	Public insurer/CEA using a model approach (decision tree)	Best ICER (€/LYG): 3,218 (4 versus 1); the exploratory surgery strategy was found to be dominated by PET	Good^b, c^

Gambhir et al. 1998, USA [99]	64-year-old white man (1.5 packs/day smoker) with a 2.5-cm nodule	(1) WW (baseline strategy); (2) Thoracotomy; (3) CT; (4) CT+PET	Public payer (Medicare)/CEA using a model approach (decision tree)	Best ICER (US$/LYG): 3,266 for CT	Good^b, c^

Gould et al. 2003, USA [100]	62-year-old patient with a new, non-calcified pulmonary nodule seen on chest radiograph	40 clinically plausible sequences of five diagnostic technologies: CT, PET, TNB, surgery, and WW (baseline strategy)	Societal/CUA using a model approach (Markov model)	Best ICER (US$/QALY): 10,935 for strategy 7 (CT: if results indeterminate, biopsy; if results benign, WW)/7,625 for strategy 7/6,515 for CT (if results indeterminate, surgery; if results benign, WW)**	Good^b^

*Staging of non-small cell lung cancer*					

Bradbury et al. 2002, UK [29]	Medically fit for either surgery or non-surgical treatment, 62-year-old patient	(1) All for surgery; (2) All for non-surgical treatment; (3) MS; (4) PET after negative MS; (5) PET; (6) MS after negative PET; (7) MS after positive PET (no N0/1 M1 disease)	Health care system/CUA using a model approach (decision tree)	Best ICER (£/QALY): 58,951 for CT-positive patients (7 versus 3); 10,475 for CT-negative patients (7 versus 1)	Good^b^

Dietlein et al. 2000, Germany [31]	62-year-old man with histologically established and assessed as locally resectable NSCLC without distant metastases	(1) Conventional staging; (2) PET in patients with normal-sized lymph nodes; (3) PET for all; (4) PET without supplementary MS if positive CT and PET; (5) PET without supplementary MS if positive PET	Public insurer/CEA using a model approach (decision tree)	Best ICER (€/LYG): 143 (2 versus 1); 15,325 (4 versus 2); 17,438 (5 versus 3)	Good^b^

Dussault et al. 2001, Canada [101]	65-year-old male with histologically confirmed NSCLC without mediastinal and distant metastases	CT+PET versus CT	Health care system/CEA using a model approach (decision tree)	ICER (C$/LYG): 4,689	Good^b^

Kosuda et al. 2000, Japan [102]	Patient with suspected NSCLC, stage IIIB or less	CT+PET versus CT	Hospital/CEA using a model approach (decision tree)	ICER (¥/LYG): 218,000	Good^b, c^

Scott et al. 1998, USA [30]	64-year-old male with NSCLC	(1) CT (MS after positive CT); (2) PET after negative CT (MS after positive CT); (3) CT+PET (MS after positive PET); (4) CT+PET (MS after positive CT or positive PET after negative CT)	Public payer (Medicare)/CEA using a model approach (decision tree)	Best ICER (US$/LYG): 25,286 (2 versus 1)	Good^c^

Sloka et al. 2004, Canada [103]	65-year-old patient with suspected NSCLC	CT+PET versus CT	Health care system/CEA using a model approach (decision tree)	Cost-savings: C$1,455^a^	Good^b^

^a^ICER not calculated on account of the clinical insignificance of the outcome difference in terms of life expectancy; ^b^Study quality was assessed by Cleemput et al. [6] using the Drummond, Jefferson checklist [9]; ^c^Study quality was assessed by Müller et al. [7] using a standardized transparency and quality catalogue [104]; *Base case; **Low/intermediate/high probability of malignancy respectively; CRC: colorectal cancer, CEA: cost-effectiveness analysis, CT: computerized tomography, CUA: cost-utility analysis, HD: Hodgkin's disease, HNSCC: head and neck squamous cell carcinoma, ICER: incremental cost-effectiveness ratio, LYG: life-year gained, MS: mediastinoscopy, ng/ml: nanograms per millilitre, NSCLC: non-small cell lung cancer, PET: positron emission tomography, QALY: quality-adjusted life-year, SPN: solitary pulmonary nodule, TNB: transthoracic needle biopsy, UK: United Kingdom, USA: United States of America, WTP: willingness to pay, WW: wait and watch.

In addition, a review of the cost-effectiveness of PET for characterizing pulmonary nodules is available from Gould [41]. Eight studies were identified that evaluated the cost-effectiveness of PET for SPN characterization. The author concluded that, despite using different methods and modelling assumptions, there was remarkable agreement across these studies. PET was found to be the most cost-effective strategy when used in patients with low to moderate pretest probability of malignancy and indeterminate nodules on CT.

### Limitations of this review

This review has several limitations. First, as the aim of this review was to undertake a systematic review of economic evaluations, a systematic review of effectiveness studies was not carried out. However, systematic reviews of effectiveness studies are considered to be the showpiece of evidence-based medicine, and the value of undertaking systematic reviews of economic evaluation studies to inform health care decision-makers is less clear. In the literature, questions remain as to whether it is actually useful to conduct reviews of existing economic evaluation studies [42]. A fundamental reason for undertaking a review of any type is that the evidence synthesis will be more useful than that available from any single study [10]. However, Anderson [43] argues that the need for systematic reviews of economic evaluations is unwarranted, because decision-analytic models are themselves a well-developed technique of evidence synthesis. However, the results of this systematic review show that not all modelling studies of oncologic PET and PET/CT were of good methodological quality. Thus, Anderson's argument can be questioned.

Second, given that the extent of the literature search was determined by the resources available to the author, sources searched for economic evaluations did not include the Health Economic Evaluations Database (HEED), a specialized database of economic evaluations. Different search methods (electronic and manual) were used, and a variety of resources were searched to conduct a comprehensive systematic review of economic evaluation studies. However, it cannot be excluded that a search in the HEED would reveal additional economic evaluations. Furthermore, experts were not contacted to identify unpublished economic evaluation studies. Therefore, publication bias cannot be excluded completely, because the full publication of studies is dependent on the direction and/or strength of their findings, and positive results are more likely to be published. In addition, the review was restricted to English, French, or German publications, and parallel independent assessments reducing the risk of errors during the study selection process were not performed.

Third, the BMJ criteria list [9] is the most general quality assessment tool [10]. However, both the study question and the inclusion criteria required a more specific quality assessment checklist. Thus, the appraisal of health economic evaluations followed the framework for the quality assessment of decision-analytic models developed by Philips et al. [11]. Given the limitations in reporting of economic evaluations, study quality was sometimes difficult to assess. Furthermore, the use of quality scoring systems is controversially discussed in the literature. In the recent Centre for Reviews and Dissemination (CRD)'s guidance of undertaking reviews in health care, their use is considered problematic and is not recommended [10]. Instead of using quality scores, a narrative critical assessment based on the Philips framework [11] was performed to reflect individual aspects of methodological quality. From the high number of quality criteria, only the most substantial methodological strengths and weaknesses were reported.

## Conclusions

### Implications for practice

Owing to improved care and less exposure to ineffective treatments, personalized medicine using PET may be cost-effective. However, the strongest evidence for the cost-effectiveness of PET is still in the staging of NSCLC. The studies suggested that PET for staging NSCLC may benefit patients in terms of a (slight) increase in life expectancy, and the health care system in terms of cost savings resulting from the number of invasive procedures avoided. Furthermore, health care deciders should consider PET as cost-effective for diagnosing solitary pulmonary nodules.

### Implications for research

Management decisions relating to the assessment of treatment response or radiotherapy treatment planning require further research to show the impact of PET on patient management and its cost-effectiveness. Owing to the potential for increased patient throughput and the possible greater accuracy, the cost-effectiveness of PET/CT may be superior to that of PET. Only four studies on the cost-effectiveness of PET/CT were found in this review, and this is clearly an area for future research. Finally, prospective trial-based economic evaluations are needed.

## Competing interests

The author declares that she has no competing interests.

## Pre-publication history

The pre-publication history for this paper can be accessed here:

http://www.biomedcentral.com/1472-6963/10/283/prepub

## Supplementary Material

Additional file 1 **Search strategies**. Documenting the search strategies used. Click here for file

Additional file 2 **Economic evaluations: Study characteristics**. Overview of study characteristics. Click here for file

Additional file 3 **Economic evaluations: Results**. Overview of study results. Click here for file

Additional file 4 **Quality assessment of economic models: Staging of NSCLC/Diagnosis of an SPN**. Quality assessment. Part I. Click here for file

Additional file 5 **Quality assessment of economic models: Staging of various cancers/metastases/Follow-up of NSCLC**. Quality assessment. Part II. Click here for file
