# Supplementary material for: A systematic review of PET and PET/CT in oncology: A way to personalize cancer treatment in a cost-effective manner?
Source: BMC Health Serv Res. 2010 Oct 8;10:283. doi: 10.1186/1472-6963-10-283 (PMC2959014; doi:10.1186/1472-6963-10-283)
Supplement: Additional file 1 — Search strategies. Documenting the search strategies used. [file 1472-6963-10-283-S1.DOC]

## Additional file 1. Search strategies

As the development of a search strategy is an iterative process, search strategies were built up from various test searches. To identify appropriate indexing terms, key papers identified from the different test searches were checked for how they were indexed. The draft strategy was subsequently tested on PubMed. The full PubMed search strategy was as follows: ("Economics"[Mesh] OR "economics"[Subheading] OR “Costs and Cost Analysis”[Mesh]) OR "Efficiency, Organizational"[Mesh] OR (cost*[ti] OR econom*[ti] OR burden of disease*[ti] OR burden of illness[ti] OR efficien*[ti]) AND ("Positron-Emission Tomography"[Mesh] OR "positron emission tomography" OR "Tomography, Emission-Computed"[Mesh] OR PET) AND ("Neoplasms"[Mesh] OR neoplasm* OR tumour* OR tumor* OR cancer* OR oncology). Eleven publications could be retrieved by using this search strategy that was adapted for the other electronic databases searched.

**The Cochrane Library** (10/02/2010): Cochrane Database of Systematic Reviews (Cochrane Reviews), Database of Abstracts of Reviews of Effects (Other Reviews), Cochrane Central Register of Controlled Trials (Clinical Trials), Cochrane Methodology Register (Methods Studies), Health Technology Assessment Database (Technology Assessments), NHS Economic Evaluation Database (Economic Evaluations), About The Cochrane Collaboration (Cochrane Groups)

| **Search** | **Query** | **Hits** |
| --- | --- | --- |
| #13 | [(#12), from 2005 to 20](http://www3.interscience.wiley.com/cochrane/searchHistory?mode=runquery&qnum=13)10 | 57 |
| #12 | [(#9 AND #10 AND #11)](http://www3.interscience.wiley.com/cochrane/searchHistory?mode=runquery&qnum=12) | 159 |
| #11 | [(#6 OR #7 OR #8)](http://www3.interscience.wiley.com/cochrane/searchHistory?mode=runquery&qnum=11) | 67,104 |
| #10 | [(#4 OR #5)](http://www3.interscience.wiley.com/cochrane/searchHistory?mode=runquery&qnum=10) | 71,368 |
| #9 | [(#1 OR #2 OR #3)](http://www3.interscience.wiley.com/cochrane/searchHistory?mode=runquery&qnum=9) | 2,859 |
| #8 | (cost*) or (econom*) or (burden of disease) or (burden of illness) or (efficien*) | 66,419 |
| #7 | [MeSH descriptor Efficiency, Organizational explode all trees](http://www3.interscience.wiley.com/cochrane/searchHistory?mode=runquery&qnum=7) | 277 |
| #6 | [MeSH descriptor Economics explode](http://www3.interscience.wiley.com/cochrane/searchHistory?mode=runquery&qnum=6) all trees | 31,659 |
| #5 | [(cancer*) or (tumour*) or (tumor*) or (oncology) or (neoplasm*)](http://www3.interscience.wiley.com/cochrane/searchHistory?mode=runquery&qnum=5) | 69,309 |
| #4 | MeSH descriptor Neoplasms explode all trees | 39,030 |
| #3 | positron emission tomography or PET | 1,973 |
| #2 | MeSH descriptor Positron-Emission Tomography explode all trees | 520 |
| #1 | MeSH descriptor Tomography, Emission-Computed explode all trees | 1,999 |

**CRD databases** (10/02/2010): Database of Abstracts of Reviews of Effects (DARE), NHS Economic Evaluation Database (NHS EED), Health Technology Assessment (HTA) Database

| **Search** | **Query** | **Hits** |
| --- | --- | --- |
| #14 | [#13 RESTRICT YR 2005 20](http://www.crd.york.ac.uk/CRDWeb/Search.aspx?SearchID=747731&SessionID=747700&D=23&E=19&H=15&SearchFor=#15%20RESTRICT%20YR%202005%202008)10 | 92 |
| #13 | #11 AND #12 | 243 |
| #12 | #5 OR #6 OR #7 OR #8 OR #9 OR #10 | 8,154 |
| #11 | #1 OR #2 OR #3 OR #4 | 428 |
| #10 | [neoplasm*](http://www.crd.york.ac.uk/CRDWeb/Search.aspx?SearchID=747718&SessionID=747700&D=94&E=1029&H=16&SearchFor= neoplasm* ) | 1,162 |
| #9 | [oncology](http://www.crd.york.ac.uk/CRDWeb/Search.aspx?SearchID=747717&SessionID=747700&D=434&E=757&H=57&SearchFor= oncology ) | 1,500 |
| #8 | [tumor*](http://www.crd.york.ac.uk/CRDWeb/Search.aspx?SearchID=747716&SessionID=747700&D=61&E=87&H=138&SearchFor= tumor* ) | 295 |
| #7 | [tumour*](http://www.crd.york.ac.uk/CRDWeb/Search.aspx?SearchID=747714&SessionID=747700&D=390&E=372&H=136&SearchFor= tumour* ) | 1,074 |
| #6 | [cancer*](http://www.crd.york.ac.uk/CRDWeb/Search.aspx?SearchID=747712&SessionID=747700&D=1710&E=2247&H=931&SearchFor= cancer* ) | 5,823 |
| #5 | [MeSH Neoplasms EXPLODE 1](http://www.crd.york.ac.uk/CRDWeb/Search.aspx?SearchID=747706&SessionID=747700&D=1148&E=2823&H=1182&SearchFor=MeSH Neoplasms EXPLODE 1) | 6,234 |
| #4 | PET | 259 |
| #3 | "positron emission tomography" | 222 |
| #2 | MeSH Tomography, Emission-Computed EXPLODE 1 2 3 4 5 | 383 |
| #1 | MeSH Positron-Emission Tomography EXPLODE 1 2 3 4 5 | 131 |

**EMBASE** (10/02/2010)

| **Search** | **Query** | **Hits** |
| --- | --- | --- |
| #4 | limit 3 to yr="2005 -Current" | 67 |
| #3 | 1 and 2 | 170 |
| #2 | positron emission tomography.af. | 46,676 |
| #1 | (cost* or econom*).ti. | 51,633 |

**PubMed** (10/02/2010)

| **Search** | **Query** | **Hits** |
| --- | --- | --- |
| #7 | (#1 OR #2 OR #3) AND #4 AND #5 Limits: Publication Date from 2005 to 2010 | 132 |
| #6 | (#1 OR #2 OR #3) AND #4 AND #5 | 368 |
| #5 | "Neoplasms"[Mesh] OR neoplasm* OR tumour* OR tumor* OR cancer* OR oncology | 2,607,467 |
| #4 | "Positron-Emission Tomography"[Mesh] OR "positron emission tomography" OR "Tomography, Emission-Computed"[Mesh] OR PET | 72,910 |
| #3 | cost*[ti] OR econom*[ti] OR burden of disease*[ti] OR burden of illness[ti] OR efficien*[ti] | 132,838 |
| #2 | "Efficiency, Organizational"[Mesh] | 13,277 |
| #1 | "Economics"[Mesh] OR "economics"[Subheading] OR "Costs and Cost Analysis"[Mesh] | 489,409 |

**RePEc** (10/02/2010)

| **Search** | **Query** | **Hits** |
| --- | --- | --- |
| #1 | positron emission tomography among working papers and articles and books & chapters and software and authors | 15 |

**Web of Science** (10/02/2010)

| **Search** | **Query** | **Hits** |
| --- | --- | --- |
| #5 | #4  Databases=SCI-EXPANDED, SSCI, A&HCI Timespan=2005-2010 | 68 |
| #4 | #1 AND #2 AND #3  Databases=SCI-EXPANDED, SSCI, A&HCI Timespan=All Years | 149 |
| #3 | Topic=(neoplasm*) OR Topic=(cancer*) OR Topic=(oncology) OR Topic=(tumor*) OR Topic=(tumour*)  Timespan=All Years. Databases=SCI-EXPANDED, SSCI, A&HCI. | >100,000 |
| #2 | Topic=(positron emission tomography) OR Topic=(PET)  Timespan=All Years. Databases=SCI-EXPANDED, SSCI, A&HCI. | 74,527 |
| #1 | Title=(cost*) OR Title=(econom*) OR Title=(burden of disease) OR Title=(burden of illness) OR Title=(efficien*)  Timespan=All Years. Databases=SCI-EXPANDED, SSCI, A&HCI. | >100,000 |
