# Supplementary material for: A systematic review of PET and PET/CT in oncology: A way to personalize cancer treatment in a cost-effective manner?
Source: BMC Health Serv Res. 2010 Oct 8;10:283. doi: 10.1186/1472-6963-10-283 (PMC2959014; doi:10.1186/1472-6963-10-283)
Supplement: Additional file 2 — Economic evaluations: Study characteristics. Overview of study characteristics. [file 1472-6963-10-283-S2.DOC]

## Additional file 2. Economic evaluations: Study characteristics

| **Cancer/management decision** | **Author, year, country** | **Comparison** | **Patient group (base case)** | **Measure of effectiveness** | **Incremental analysis** | **Method, perspective** |
| --- | --- | --- | --- | --- | --- | --- |
| Staging of breast cancer | Sloka et al. 2005, Canada | a) ALND in all patients (MRM or one- or two-stage BCS+ALND+RT)  b) PET with ALND in selected patients (If PET+ then MRM or one- or two-stage BCS+ALND+RT; If PET- then MRM or BCS+RT) | 55-year-old woman presenting with stage I or II breast cancer | Increase in life expectancy | Cost-effectiveness | Decision tree,  Hospital |
| Staging of recurrent ovarian cancer | Mansueto et al. 2009, Italy | a) CT (If CT+ and single/ multiple lesion then surgery/ chemotherapy; If CT- then follow-up)  b) PET/CT for CT- (If PET/CT+ and single/ multiple lesion then surgery/ chemotherapy, If PET/CT- then follow-up)  c) PET/CT for all (See b) | Thirty-two consecutive patients (mean age: 57.3 years; range: 39–75 years), with suspected ovarian cancer recurrence | Surgery avoided | Cost-effectiveness | Decision tree,  Health care system |
| Staging of head and neck cancer | Sher et al. 2009, USA | a) ND for all (baseline strategy)  b) ND for patients with RD on CT  c) ND for patients with RD on PET/CT | 50-year-old man with node-positive stage IVA (i.e. T1-3 N2 M0) SCC of the oropharynx | QALY | Cost-utility | Markov model,  Medicare? |
| Staging of recurrent nasopharyngeal carcinoma (NPC) | Yen et al. 2009, Taiwan | a) MRI (baseline strategy)  b) PET  c) MRI-PET (performing PET if MRI uncertain) | 46-year-old male patient who was suspected of having recurrent NPC during post-treatment follow-up | QALY | Cost-utility | Decision tree,  Health care system? |
| Staging of pulmonary metastases from malignant melanoma | Krug et al. 2010, Belgium | a) PET/CT  b) WB CT | Patients with resected high risk MM (stage IIc and III) | LMG | Cost-effectiveness | Markov model,  Health care system |
| Staging of liver metastases from colorectal cancer | Lejeune et al. 2005, France | a) CT (If CT+ then biopsy, followed by a second biopsy if negative results; If CT- then MRI)  b) CT+PET (If CT+ then biopsy, followed by a second biopsy if negative results (If liver biopsy+ then PET); If CT- then PET) | 68-year-old patient who had previously undergone resection for CRC, with suspected metachronous liver metastases, which were defined as lesions diagnosed during post-treatment follow-up by abdominal ultrasonography | LYG | Cost-effectiveness | Decision tree,  Health care system |
| Follow-up of non-small cell lung cancer (NSCLC) | Van Loon et al. 2010, The Netherlands | a) Conventional follow-up (anamnesis, physical examination and a chest X-ray)  b) CT-based follow-up (anamnesis, physical examination and a chest CT scan)  c) PET/CT-based follow-up (anamnesis, physical examination and a WB PET/CT scan) | NSCLC patients after radical RT with or without chemotherapy | QALY | Cost-utility | Markov model,  Health care system |

## Additional file 2. Economic evaluations: Study characteristics (cont’d)

| **Cancer/management decision** | **Author, year, country** | **Comparison** | **Patient group (base case)** | **Measure of effectiveness** | **Incremental analysis** | **Method, perspective** |
| --- | --- | --- | --- | --- | --- | --- |
| Staging of non-small cell lung cancer (NSCLC) | Alzahouri et al. 2005, France | a) Chest CT (If CT+ then MS or chemo-radiation; If CT- then surgery)  b) Chest PET for CT- (If CT+ then MS or chemo-radiation; If CT- then PET)  c) Chest PET for all with anatomical (chest) CT (If PET+ then MS or chemo-radiation; If PET- then surgery)  d) Chest CT + chest PET for all (If CT+ and PET+ then chemo-radiation; If CT+ and PET- then MS or chemo-radiation; If CT- and PET+ then MS or chemo-radiation; If CT- and PET- then surgery) | 65-year-old patient without distant metastases, in whom NSCLC has been histologically established and assessed as locally resectable by conventional staging | LYG | Cost-effectiveness | Decision tree,  Health care system |
|  | Bird et al. 2007, Australia | a) CWU consisting of an x-ray, a chest CT scan and brochoscopy (If CT- then MS or surgery; If CT+ then MS)  b) CWU + WB PET (If PET+ then MS; If PET- then thoracotomy; If PET+ for distant metastasis then palliative care) | 62-year-old man with whom NSCLC had been confirmed and who was fit for surgery or non surgical treatment. Distant metastasis had not been detected by conventional staging | QALY | Cost-utility | Decision tree,  Societal |
|  | Kee et al. 2010, UK | a) All patients will undergo MS (If MS- then surgery; If MS+ then chemo-radiation)  b) All patients have a WB PET scan (If PET- then surgery; If PET+ then chemo-radiation) | Patient with pathologically confirmed NSCLC (CT scan±bronchoscopy) referred for surgery | QALY | Cost-utility | Markov model,  Health care system? |
|  | Mansueto et al. 2007, Italy | a) CT  b) WB PET for indefinite CT  c) WB PET for all  For all 3 strategies: If CT or PET+ then medical therapy or surgery, while if CT or PET indefinite then diagnostic surgery, further diagnostic investigation or WW | Patients with known or suspected lung cancer | LYG | Cost-effectiveness | Decision tree,  Health care system |

## Additional file 2. Economic evaluations: Study characteristics (cont’d)

| **Cancer/management decision** | **Author, year, country** | **Comparison** | **Patient group (base case)** | **Measure of effectiveness** | **Incremental analysis** | **Method, perspective** |
| --- | --- | --- | --- | --- | --- | --- |
| Staging of non-small cell lung cancer (NSCLC) | National Collaborating Centre for Acute Care, 2005, UK, Surgery model | a) Patients go straight to thoracotomy  b) Patients have a MS and then receive either radical RT (MS=N2/3) or thoracotomy (MS=N0/1)  c) Patients have a PET scan and then receive either ASC (PET=M1+) or thoracotomy (PET=M0 N0/1) or go on to MS (PET=M0 N2/3) | Patients with potentially operable NSCLC (normal-sized lymph nodes on CT) with no clinical evidence of distant metastasis | QALY | Cost-utility | Decision tree,  Health care system |
|  | National Collaborating Centre for Acute Care, 2005, UK, RT model | a) Patients go straight to radical RT  b) Patients have a PET scan and then receive either ASC (PET=M1+) or thoracotomy (PET=M0 N0/1) or radical RT (PET=M0 N2/3) | Patients with proven NSCLC and no clinical evidence of distant metastasis, who have a good performance status permitting radical RT, but who are not suitable for surgery or have refused surgery | QALY | Cost-utility | Decision tree,  Health care system |
|  | Nguyen et al. 2005, Canada | a) Chest CT: If CT+ then MS; If CT- then surgery  b) Chest CT+WB PET: If CT+ then PET limited to detecting distant metastases (Biopsy or MS to confirm PET results); If CT- then PET for detecting mediastinal and distant metastases (Biopsy or MS to confirm PET results except for PET- for medistinal and distant metastases then surgery) | Medically fit-for-surgery 65-year-old man with histologically confirmed NSCLC in whom pre-operative staging using conventional detection techniques was negative for mediastinal and distant metastases | LYG | Cost-effectiveness | Decision tree,  Health care system |
| Diagnosis of a solitary pulmonary nodule (SPN) | Lejeune et al. 2005, France | a) Wait and watch  b) PET (If PET+ then biopsy or surgery; If PET- then WW)  c) CT+PET (If CT+ and PET+ then surgery; If CT+ and PET- then biopsy; If CT- and PET+ then biopsy; If CT- and PET- then WW) | 65-year-old male current smoker (1.5 packs per day) with a 2-cm SPN without calcification, specula and enlargements of mediastinal lymph nodes | LYG | Cost-effectiveness | Decision model with two components: Decision tree and Markov model,  Health care system |
| ALND: axillary lymph node dissection, ASC: active supportive care, BCS: breast conserving surgery, CRC: colorectal cancer, CT: computed tomography, CWU: conventional work up, LMG: life months gained, LYG: life years gained, MM: malignant melanoma, MRI: magnetic resonance imaging, MRM: modified radical mastectomy, MS: mediastinoscopy, ND: neck dissection, NPC: nasopharyngeal carcinoma, NSCLC: non-small cell lung cancer, PET: positron emission tomography, QALY: quality adjusted life year, RD: residual disease, RT: radiotherapy, SCC: squamous cell carcinoma, SPN: solitary pulmonary nodule, UK: United Kingdom, USA: United States of America, WB: whole body, WW: wait and watch | | | | | | |
