# Supplementary material for: A systematic review of PET and PET/CT in oncology: A way to personalize cancer treatment in a cost-effective manner?
Source: BMC Health Serv Res. 2010 Oct 8;10:283. doi: 10.1186/1472-6963-10-283 (PMC2959014; doi:10.1186/1472-6963-10-283)
Supplement: Additional file 3 — Economic evaluations: Results. Overview of study results. [file 1472-6963-10-283-S3.DOC]

## Additional file 3. Economic evaluations: Results

| **Cancer/ management decision** | **Author, year, country** | **Comparison** | **Effectiveness (per patient)** | **Cost (per patient)** | **Incremental cost-effectiveness** | **Sensitivity analysis** |
| --- | --- | --- | --- | --- | --- | --- |
| Staging of breast cancer | Sloka et al. 2005, Canada | a) ALND in all patients  b) PET with ALND in selected patients | Reported in aggregated form only: Compared with a), b) resulted in an increase in life expectancy of 7.4 days | a) C$9,178  b) C$8,483 | b) dominates a) | The SA revealed that the cost-savings remained in favour of b) if:  PET cost <C$1,724  PET specificity >49.7%  Prevalence of node positivity <50.1%  Patient selection of BCS >1.2%. For any value of PET sensitivity, b) remained dominant |
| Diagnosis of recurrent ovarian cancer | Mansueto et al. 2009, Italy | a) CT  b) PET/CT for CT-  c) PET/CT for all | Number of surgeries:  a) 15  b) 20  c) 12 | a) €2,228  b) €2,957  c) €2,909 | a) dominates b)  c) vs. a) €227/ surgery avoided | Both in the one-way and multivariate SA, results were favourable to c). The ICER varied between €91 and €379 in the one-way SA, and between €50 and €433 in the multivariate SA |
| Determining the need for adjuvant neck dissection in locally advanced head and neck cancer | Sher et al. 2009, USA | a) ND for all patients  b) ND for patients with RD on CT  c) ND for patients with RD on PET/CT | Not reported | Not reported | c) was the dominant strategy | c) remained the dominant strategy over a wide range of assumptions |
| Diagnosis of recurrent nasopharyngeal carcinoma (NPC) | Yen et al. 2009, Taiwan | a) MRI  b) PET  c) MRI-PET | a) 16.16 QALYs  b) 16.70 QALYs  c) 17.35 QALYs | a) US$350  b) US$1,100  c) US$900 | b) vs. a) US$1,389/ QALY  c) vs. a) US$462/ QALY | The SA revealed that c) remained cost-effective if: Cost ratio of PET/MRI >1.85 and probability of uncertain MRI <73%, respectively |
| Staging of pulmonary metastases from malignant melanoma | Krug et al. 2010, Belgium | a) PET/CT  b) CT | a) 90.61 LMG  b) 90.42 LMG | a) €3,438  b) €4,384 | a) dominates b) | The acceptability curve shows that 71% of trials are dominant and 6.4% have a high cost-effectiveness with a low incremental cost for a) |
| Staging of liver metastases from colorectal cancer | Lejeune et al. 2005, France | a) CT  b) CT+PET | a) 1.88 LYs  b) 1.88 LYs | a) €19,735  b) €17,064 | b) dominates a) | Only when the cost of PET was set at €8,992 did CT become the preferred option |
| Follow-up of non-small cell lung cancer (NSCLC) | Van Loon et al. 2010, The Netherlands | a) Conventional follow-up  b) CT-based follow-up  c) PET/CT-based follow-up | For all patients:  a) 1.28 QALYs  b) 1.28 QALYs  c) 1.30 QALYs | a) €13,983  b) €14,269  c) €15,266 | b) vs. a) €264,033/ QALY  c) vs. a) €69,086/ QALY | Given a WTP per QALY gained of €80,000, a) and c) had a similar probability of being cost-effective (47% and 48%, respectively), while the probability of b) being cost-effective was only 5% |

## Additional file 3. Economic evaluations: Results (cont’d)

| **Cancer/ management decision** | **Author, year, country** | **Comparison** | **Effectiveness (per patient)** | **Cost (per patient)** | **Incremental cost-effectiveness** | **Sensitivity analysis** |
| --- | --- | --- | --- | --- | --- | --- |
| Staging of non-small cell lung cancer (NSCLC) | Alzahouri et al. 2005, France | a) CT  b) PET for CT-  c) PET for all with anatomical CT  d) CT+PET for all | a) 3.47 LYs  b) 3.49 LYs  c) 3.57 LYs  d) 3.44 LYs | a) €4,542  b) €5,206  c) €4,481  d) €5,550 | b) vs. a) €33,165/ LYG  c) dominates a)  a) dominates d) | If frequency of biopsy 0<p<0.5, c) is the dominant strategy;  If PET Sp/CT- <73%, a) is the dominant strategy;  If PET Sp/CT- >73%, c) is the most cost-effective strategy |
|  | Bird et al. 2007, Australia | a) CWU  b) CWU+PET | CT- patients:  a) 2.88 QALYs  b) 2.91 QALYs  CT+ patients:  a) 2.09 QALYs  b) 2.11 QALYs | CT- patients:  a) A$20,427  b) A$20,826  CT+ patients:  a) A$23,578  b) A$24,083 | CT- patients:  b) vs. a) A$14,581/ QALY gained  CT+ patients:  b) vs. a) A$52,039/ QALY gained | CT- patients: The ICER stayed within a range of A$11,205 (increasing upstaging in N2/3 patients during surgery by 50%) to A$19,268 (surgical morbidity decreased to 0.1) or b) was dominant.  CT+ patients: There was much uncertainty surrounding the base result |
|  | Kee et al. 2010, UK | a) MS  b) PET | For a 50/60/70/80-year old:  a) 2.3129/ 2.0450/ 1.7450/ 1.4267 QALYs  b) 2.3377/ 2.0648/ 1.7607/ 1.4388 QALYs | For all 4 age groups:  a) £4,827  b) £4,994 | For a 50/60/70/80-year old:  b) vs. a) (£/ QALY gained) 6,704/ 8,385/ 10,636/ 13,785 | The EVPI associated with the patient related utility of a futile thoracotomy considerably exceeded that associated with the accuracy of PET and CT, respectively |
|  | Mansueto et al. 2007, Italy | a) CT  b) PET for indefinite CT  c) PET for all | a) 1.96 LYs  b) 2.04 LYs  c) 2.64 LYs | a) €2,535  b) €2,735  c) €2,985 | b) vs. a) €2,508/ LYG  c) vs. b) €415/ LYG | Over a wide range of assumptions, c) remained the most cost-effective strategy |

## Additional file 3. Economic evaluations: Results (cont’d)

| **Cancer/ management decision** | **Author, year, country** | **Comparison** | **Effectiveness (per patient)** | **Cost (per patient)** | **Incremental cost-effectiveness** | **Sensitivity analysis** |
| --- | --- | --- | --- | --- | --- | --- |
| Staging of non-small cell lung cancer (NSCLC) | National Collaborating Centre for Acute Care, 2005, UK, Surgery model | a) Thoracotomy  b) MS  c) PET | Base case results for 100 potentially patients being considered for surgery:  a) 284.6 QALYs  b) 286.4 QALYs  c) 288.3 QALYs | Base case results for 100 potentially patients being considered for surgery:  a) £631,739  b) £751,971  c) £658,187 | c) dominates b)  c) vs. a) £7,199/ QALY gained | Only when the cost of PET is high and the cost of thoracotomy low does the ICER exceed £30,000/ QALY. c) could actually be cost saving overall compared with a) if the cost of PET were to fall or if the population scanned had a particularly high prevalence of distant metastases. c) was cost saving compared with b), except when the cost of MS was low relative to the cost of PET |
|  | National Collaborating Centre for Acute Care, 2005, UK, RT model | a) Radical RT  b) PET | Base case results for 100 patients being considered for radical RT:  a) 84.5 QALYs  b) 88.8 QALYs | Base case results for 100 patients being considered for radical RT:  a) £760,600  b) £801,536 | b) vs. a) £9,489/ QALY gained | Only when the cost of PET exceeds the cost of radical RT or when radical RT is more effective than surgery does the ICER exceed £30,000/ QALY. b) could actually be cost saving overall compared with a) when the cost of radical RT is high relative to the cost of PET and palliative RT |
|  | Nguyen et al. 2005, Canada | a) CT  b) CT+PET | a) 4.551 LYs  b) 4.823 LYs | a) C$8,455  b) C$9,723 | b) vs. a) C$4,689/ LYG | The ICER ranged from C$3,000 to C$5,000/ LYG. In 95% of the Monte Carlo simulations, the cost per LYG was less than C$50,000 |
| Diagnosis of a solitary pulmonary nodule (SPN) | Lejeune et al. 2005, France | a) Wait and watch  b) PET  c) CT+PET | a) 12.81 LYs  b) 13.73 LYs  c) 13.78 LYs | a) €6,327  b) €8,770  c) €7,959 | b) vs. a) €4,790/ LYG  c) vs. a) €3,022/ LYG | The results of the SA showed that c) remained the most cost-effective strategy when the risk of SPN malignancy was in the range of 5.7 to 87%, whereas a) was more cost-effective in the range of 0.3 to 5% |
| ALND: axillary lymph node dissection, BCS: breast conserving surgery, CT: computed tomography, CWU: conventional work up, EVPI: expected value of perfect information, ICER: incremental cost-effectiveness ratio, LMG: life months gained, LYG: life year gained, LYs: life years, MRI: magnetic resonance imaging, MS: mediastinoscopy, ND: neck dissection, NPC: nasopharyngeal carcinoma, NSCLC: non-small cell lung cancer, p: probability, PET: positron emission tomography, QALY(s): quality adjusted life year(s), RD: residual disease, RT: radiotherapy, SA: sensitivity analysis, Sp: specificity, SPN: solitary pulmonary nodule, UK: United Kingdom, USA: United States of America, WTP: willingness to pay | | | | | | |
