# Supplementary material for: A systematic review of PET and PET/CT in oncology: A way to personalize cancer treatment in a cost-effective manner?
Source: BMC Health Serv Res. 2010 Oct 8;10:283. doi: 10.1186/1472-6963-10-283 (PMC2959014; doi:10.1186/1472-6963-10-283)
Supplement: Additional file 5 — Quality assessment of economic models: Staging of various cancers/metastases/Follow-up of NSCLC. Quality assessment. Part II. [file 1472-6963-10-283-S5.DOC]

## Additional file 5. Quality assessment of economic models: Staging of various cancers/metastases/follow-up of NSCLC

| **Dimension of quality** | | **Staging of** | | | | | | **Follow-up of** |
| --- | --- | --- | --- | --- | --- | --- | --- | --- |
|  |  | *Breast cancer* | *Recurrent ovarian cancer* | *Head and neck cancer* | *Recurrent nasopharyngeal carcinoma* | *Pulmonary metastases from malignant melanoma* | *Liver metastases from colorectal cancer* | *NSCLC* |
| *Quality criteria* | *Question(s) for critical appraisal*  0 = No, 1 = Yes, ? = Unclear, NA = Not applicable | *Sloka et al. 2005* | *Mansueto et al. 2009* | *Sher et al. 2009* | *Yen et al. 2009* | *Krug et al. 2010* | *Lejeune et al. 2005* | *Van Loon et al. 2010* |
| **Structure** | | | | | | | | |
| **S1** Statement of decision problem/ objective | Is there a clear statement of the decision problem? | 1 | 1 | 1 | 1 | 1 | 1 | 1 |
|  | Is the objective of the evaluation and model specified and consistent with the stated decision problem? | 1 | 1 | 1 | 1 | 1 | 1 | 1 |
|  | Is the primary decision maker specified? | 0 | 0 | 0 | 0 | 0 | 0 | 0 |
| **S2** Statement of scope/ perspective | Is the perspective of the model stated clearly? | 1 | 1 | 0 | 0 | 1 | 1 | 1 |
|  | Are the model inputs consistent with the stated perspective? | 1 | 1 | NA | NA | 1 | 1 | 1 |
|  | Has the scope of the model been stated and justified? | 1 | 1 | 1 | 1 | 1 | 1 | 1 |
|  | Are the outcomes of the model consistent with the perspective, scope and overall objective of the model? | 1 | 1 | 1 | 1 | 1 | 1 | 1 |
| **S3** Rationale for structure | Is the structure of the model consistent with a coherent theory of the health condition under evaluation? | 0 | 0 | 1 | 0 | 1 | 0 | 1 |
|  | Are the sources of data used to develop the structure of the model specified? | 1 | 0 | 0 | 0 | 0 | 1 | 1 |
|  | Are the causal relationships described by the model structure justified appropriately? | 0 | 0 | 0 | 0 | 0 | 1 | 0 |
| **S4** Structural assumptions | Are the structural assumptions transparent and justified? | 1 | 1 | 1 | 1 | 1 | 1 | 1 |
|  | Are the structural assumptions reasonable given the overall objective, perspective and scope of the model? | 1 | 1 | 1 | 0 | 1 | 1 | 1 |
| **S5** Strategies/ comparators | Is there a clear definition of the options under evaluation? | 1 | 1 | 1 | 0 | 1 | 1 | 1 |
|  | Have all feasible and practical options been evaluated? | 0 | 1 | 1 | ? | 1 | 1 | 1 |
|  | Is there justification for the exclusion of feasible options? | 0 | NA | NA | NA | NA | 1 | NA |
| **S6** Model type | Is the chosen model type appropriate given the decision problem and specified causal relationships within the model? | 1 | 1 | 1 | 1 | 1 | 1 | 1 |
| **S7** Time horizon | Is the time horizon of the model sufficient to reflect all important differences between options? | 0 | 1 | 1 | 0 | 1 | 1 | 1 |
|  | Are the time horizon of the model, the duration of treatment and the duration of treatment effect described and justified? | ?/NA/NA | 0/NA/NA | 1/NA/NA | 0/NA/NA | 1/NA/NA | 1/NA/NA | 1/NA/NA |

## Additional file 5. Quality assessment of economic models: Staging of various cancers/metastases/follow-up of NSCLC (cont’d)

| **Dimension of quality** | | **Staging of** | | | | | | **Follow-up of** |
| --- | --- | --- | --- | --- | --- | --- | --- | --- |
|  |  | *Breast cancer* | *Recurrent ovarian cancer* | *Head and neck cancer* | *Recurrent nasopharyngeal carcinoma* | *Pulmonary metastases from malignant melanoma* | *Liver metastases from colorectal cancer* | *NSCLC* |
| *Quality criteria* | *Question(s) for critical appraisal*  0 = No, 1 = Yes, ? = Unclear, NA = Not applicable | *Sloka et al. 2005* | *Mansueto et al. 2009* | *Sher et al. 2009* | *Yen et al. 2009* | *Krug et al. 2010* | *Lejeune et al. 2005* | *Van Loon et al. 2010* |
| **Structure (cont’d)** | | | | | | | | |
| **S8** Disease states/ pathways | Do the disease states (state transition model) or the pathways (decision tree model) reflect the underlying biological process of the disease in question and the impact of interventions? | 0 | 0 | 1 | 0 | 1 | 0 | 1 |
| **S9** Cycle length | Is the cycle length defined and justified in terms of the natural history of disease? | NA | NA | 0 | NA | 1 | NA | 1 |
| **Data** | | | | | | | | |
| **D1** Data identification | Are the data identification methods transparent and appropriate given the objectives of the model? | 1 | 0 | 0 | 0 | 0 | 0 | 0 |
|  | Where choices have been made between data sources, are these justified appropriately? | 1 | NA | 0 | 0 | 0 | 0 | NA |
|  | Has particular attention been paid to identifying data for the important parameters in the model? | 0 | 0 | 0 | 0 | 0 | 0 | 0 |
|  | Has the quality of the data been assessed appropriately? | 0 | 0 | 0 | 0 | 0 | 1 | 0 |
|  | Where expert opinion has been used, are the methods described and justified? | NA | NA | NA | 0 | 0 | 0 | 0 |
| **D2** Pre-model data analysis | Is the data modelling methodology based on justifiable statistical and epidemiological techniques? | 0 | 0 | 0 | 0 | 0 | 0 | 0 |
| **D2a** Baseline data | Is the choice of baseline data described and justified? | 1 | 0 | 1 | 1 | 1 | 1 | 1 |
|  | Are transition probabilities calculated appropriately? | NA | NA | 0 | NA | 0 | NA | 0 |
|  | Has a half cycle correction been applied to both cost and outcome? | NA | NA | 0 | NA | 0 | NA | 0 |
|  | If not, has this omission been justified? | NA | NA | 0 | NA | 0 | NA | 0 |

## Additional file 5. Quality assessment of economic models: Staging of various cancers/metastases/follow-up of NSCLC (cont’d)

| **Dimension of quality** | | **Staging of** | | | | | | **Follow-up of** |
| --- | --- | --- | --- | --- | --- | --- | --- | --- |
|  |  | *Breast cancer* | *Recurrent ovarian cancer* | *Head and neck cancer* | *Recurrent nasopharyngeal carcinoma* | *Pulmonary metastases from malignant melanoma* | *Liver metastases from colorectal cancer* | *NSCLC* |
| *Quality criteria* | *Question(s) for critical appraisal*  0 = No, 1 = Yes, ? = Unclear, NA = Not applicable | *Sloka et al. 2005* | *Mansueto et al. 2009* | *Sher et al. 2009* | *Yen et al. 2009* | *Krug et al. 2010* | *Lejeune et al. 2005* | *Van Loon et al. 2010* |
| **Data (cont’d)** | | | | | | | | |
| **D2b** Treatment effects | If relative treatment effects have been derived from trial data, have they been synthesised using appropriate techniques? | NA | NA | NA | NA | NA | NA | NA |
|  | Have the methods and assumptions used to extrapolate short-term results to final outcomes been documented and justified? | NA | NA | NA | NA | NA | NA | NA |
|  | Have alternative extrapolation assumptions been explored through sensitivity analysis? | NA | NA | NA | NA | NA | NA | NA |
|  | Have assumptions regarding the continuing effect of treatment once treatment is complete been documented and justified? | NA | NA | NA | NA | NA | NA | NA |
|  | Have alternative assumptions regarding the continuing effect of treatment been explored through sensitivity analysis? | NA | NA | NA | NA | NA | NA | NA |
| **D2c** Costs | Are the costs incorporated into the model justified? | 1 | 1 | 1 | 0 | 1 | 1 | 1 |
|  | Has the source for all costs been described? | 1 | 1 | 1 | 1 | 1 | 1 | 1 |
|  | Have discount rates been described and justified given the target decision-maker? | 0 | 0 | 1 | 0 | 1 | 1 | 1 |
| **D2d** Quality-of-life weights (utilities) | Are the utilities incorporated into the model appropriate? | NA | NA | 1 | 1 | NA | NA | 1 |
|  | Is the source for the utility weights referenced? | NA | NA | 1 | 1 | NA | NA | 1 |
|  | Are the methods of derivation for the utility weights justified? | NA | NA | 0 | 0 | NA | NA | 0 |
| **D3** Data incorporation | Have all data incorporated into the model been described and referenced in sufficient detail? | 1 | 1 | 1 | 0 | 1 | 1 | 1 |
|  | Has the use of mutually inconsistent data been justified (i.e. are assumptions and choices appropriate)? | NA | NA | NA | NA | NA | NA | NA |
|  | Is the process of data incorporation transparent? | 1 | 1 | 1 | 1 | 1 | 1 | 0 |
|  | If data have been incorporated as distributions, has the choice of distribution for each parameter been described and justified? | NA | NA | 1 | NA | 1 | NA | 1 |
|  | If data have been incorporated as distributions, is it clear that second order uncertainty is reflected? | NA | NA | 0 | NA | 0 | NA | 0 |

## Additional file 5. Quality assessment of economic models: Staging of various cancers/metastases/follow-up of NSCLC (cont’d)

| **Dimension of quality** | | **Staging of** | | | | | | **Follow-up of** |
| --- | --- | --- | --- | --- | --- | --- | --- | --- |
|  |  | *Breast cancer* | *Recurrent ovarian cancer* | *Head and neck cancer* | *Recurrent nasopharyngeal carcinoma* | *Pulmonary metastases from malignant melanoma* | *Liver metastases from colorectal cancer* | *NSCLC* |
| *Quality criteria* | *Question(s) for critical appraisal*  0 = No, 1 = Yes, ? = Unclear, NA = Not applicable | *Sloka et al. 2005* | *Mansueto et al. 2009* | *Sher et al. 2009* | *Yen et al. 2009* | *Krug et al. 2010* | *Lejeune et al. 2005* | *Van Loon et al. 2010* |
| **Data (cont’d)** | | | | | | | | |
| **D4** Assessment of uncertainty | Have the four principal types of uncertainty been addressed? | 0 | 0 | 0 | 0 | 0 | 0 | 0 |
|  | If not, has the omission of particular forms of uncertainty been justified? | 0 | 0 | 0 | 0 | 0 | 0 | 0 |
| **D4a** Methodological | Have methodological uncertainties been addressed by running alternative versions of the model with different methodological assumptions? | 0 | 0 | 0 | 0 | 0 | 0 | 0 |
| **D4b** Structural | Is there evidence that structural uncertainties have been addressed via sensitivity analysis? | 1 | 0 | 0 | 0 | 0 | 0 | 0 |
| **D4c** Heterogeneity | Has heterogeneity been dealt with by running the model separately for different sub-groups? | 0 | 0 | 0 | 0 | 0 | 0 | 1 |
| **D4d** Parameter | Are the methods of assessment of parameter uncertainty appropriate? | 0 | 0 | 1 | 0 | 1 | 0 | 1 |
|  | If data are incorporated as point estimates, are the ranges used for sensitivity analysis stated clearly and justified? | 0 | 1 | 1 | 1 | 1 | 1 | NA |
| **Consistency** | | | | | | | | |
| **C1** Internal consistency | Is there evidence that the mathematical logic of the model has been tested thoroughly before use? | 0 | 0 | 0 | 0 | 0 | 0 | 0 |
| **C2** External consistency | Are any counterintuitive results from the model explained and justified? | NA | NA | NA | NA | NA | NA | NA |
|  | If the model has been calibrated against independent data, have any differences been explained and justified? | NA | NA | 1 | NA | NA | NA | NA |
|  | Have the results of the model been compared with those of previous models and any differences in results explained? | 0 | 0 | 0 | 0 | 1 | 1 | 0 |
